# Postoperative immune-related adverse events after conversion surgery following induction immune checkpoint inhibitor-combined chemotherapy in patients with unresectable esophagogastric junction carcinoma: a report of two cases

**DOI:** 10.1186/s13019-025-03777-0

**Published:** 2025-12-31

**Authors:** Rie Nakashima, Kazuo Koyanagi, Miho Yamamoto, Akihito Kazuno, Yoshiaki Shoji, Yamato Ninomiya, Kohei Kanamori, Kohei Tajima, Mika Ogimi, Takayuki Nishi, Masaki Mori

**Affiliations:** Department of Gastroenterological Surgery, Tokai University School of Medicine, 143 Shimokasuya, Isehara, 259-1193 Kanagawa Japan

**Keywords:** Immune-related adverse event, Esophagogastric junction cancer, Conversion surgery, Immune checkpoint inhibitor

## Abstract

**Background:**

An increasing number of patients are undergoing conversion surgery owing to the rising popularity of relatively strong regimens such as immune checkpoint inhibitor (ICI)-combined chemotherapy for the treatment of unresectable esophageal or esophagogastric junction (EGJ) cancer. However, the perioperative safety of conversion surgery after ICI combined with chemotherapy remains unclear. We report two cases of postoperative immune-related adverse events (irAEs) in patients who underwent conversion surgery after the induction of ICI-combined chemotherapy.

**Case presentations:**

Case 1: A patient with unresectable EGJ adenocarcinoma and para-abdominal aortic lymph node (LN) metastasis developed adrenal insufficiency after four courses of nivolumab + S1 + oxaliplatin (Nivo+ SOX) therapy. As significant tumor shrinkage was observed, conversion surgery was performed. While the postoperative course was uneventful, two months after surgery, hypothyroidism developed and was determined to be an irAE. Thyroid hormone replacement therapy was administered and the symptoms improved.

Case 2: A patient with EGJ adenocarcinoma and extensive metastasis to mediastinal, supraclavicular, and para-aortic LNs, along with aortic invasion, also received four courses of Nivo + SOX. Hypothyroidism developed during treatment and was managed with thyroid hormone replacement therapy. Following notable tumor regression, the patient underwent conversion surgery. The patient’s postoperative course was uneventful and was discharged. However, one month after surgery, the patient was readmitted with severe respiratory distress and was diagnosed with interstitial pneumonia. Intensive care with extracorporeal membrane oxygenation (ECMO) was initiated following steroid and endoxan pulse therapy, but the patient died four months after surgery.

****Conclusions**:**

The risk of irAEs should always be considered in patients receiving ICIs, even after conversion surgery. This condition should be differentiated from complications of esophagectomy and managed promptly.

## Background

Immune checkpoint inhibitor (ICI)-combined chemotherapy has become the standard initial treatment for patients with locally advanced or metastatic unresectable esophageal and esophagogastric junction (EGJ) cancers. ICIs can lead to marked tumor downstaging, enabling conversion surgery in selected patients [1]. Although previous reports suggested that conversion surgery might contribute to survival after ICI combined chemotherapy [2], little is known about its perioperative safety. Herein, we present two cases of immune-related adverse events (irAEs) that developed after conversion surgery in patients administered preoperative ICI-combined chemotherapy.

## Case presentations

### Case 1

**Fig. 1:**
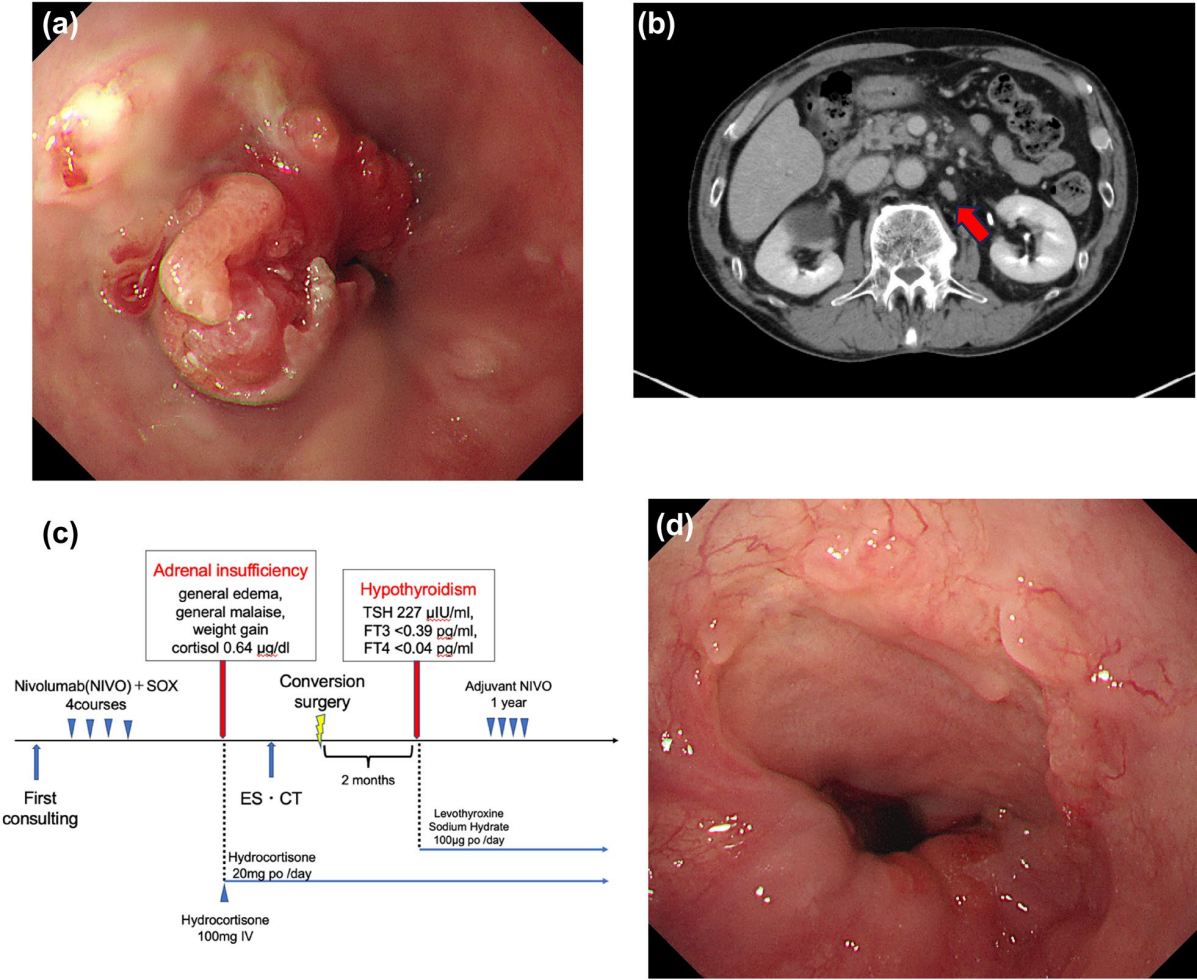
Imaging for Case 1: Initial EGD showing a Type 2 lesion around the EGJ with 3 cm esophageal invasion (**a**). Enhanced CT demonstrating #16 LNs (**b**). Treatment course (**c**). EGD showing marked shrinkage of the primary tumor (**d**) following induction of ICI-combined chemotherapy. EGD, esophagogastroduodenoscopy; EGJ, esophagogastric junction; CT, computed tomography; LN, lymph node; ICI, immune checkpoint inhibitor

A 77-year-old male with a history of prostate cancer who remained recurrence-free after radiation therapy presented with a sour stomach. Esophagogastroduodenoscopy (EGD) revealed a Siewert type II tumor around the EGJ with 3 cm of esophageal invasion (Fig. 1a). Histopathological examination of a biopsy specimen led to a diagnosis of adenocarcinoma, and the human epidermal growth factor receptor 2 (HER2)-negative due to HER2-immunohistochemistry (IHC) 2+/fluorescence in situ hybridization (FISH) -. Computed tomography (CT) revealed thickening of the wall from the lower thoracic esophagus to the EGJ and enlarged regional lymph nodes (LNs) (LN stations #3, #7: Japanese Classification of Esophageal Cancer, 12th Edition [3]) and para-abdominal aortic LNs (LN station #16) (Fig. 1b). The clinical diagnoses of the tumors were cT3, N1 (#3, #7), M1 (#16), and Stage IVB, according to 8th edition of the Union for International Cancer Control (UICC) TNM Classification of Malignant Tumors (UICC 8th edition [4]).

The standard first-line treatment for HER2-negative unresectable advanced EGJ cancer in Japan, nivolumab + S1 + oxaliplatin (Nivo + SOX), was administered (Fig. 1c). After four courses of Nivo + SOX, the patient developed systemic edema, fatigue, and significant weight gain. The blood test results showed low levels of cortisol at 0.64 µg/dL (normal range: 7.07–19.6) and adrenocorticotropic hormone (ACTH) at 2.4 pg/mL (normal range: 7.2–63.3). The patient was subsequently diagnosed with adrenal insufficiency due to irAEs, which was treated with steroid replacement therapy as directed by an endocrinologist. Imaging examination performed after confirming the absence of symptom relapse due to reduced steroid hormone showed marked shrinkage of both the primary tumor and regional LNs, indicating the treatment efficacy of Nivo + SOX (Fig. 1d). Therefore, conversion surgery was planned and Ivor Lewis procedure along with sampling of the para-abdominal aortic LNs was safely performed. The operation time was 513 min, with an intraoperative blood loss of 175 mL.

The postoperative course was uneventful, and the patient was discharged on postoperative day 12. The pathological diagnosis was ypT2N0M0, Stage ⅡA (UICC 8th ed.), with a histological therapeutic effect of grade 2 (Japanese Classification of Esophageal Cancer 12th ed.).

Two months after conversion surgery, the patient experienced fatigue and systemic edema. The blood test results showed high level of thyroid-stimulating hormone (TSH) at 227 µIU/mL (normal range: 0.61–4.23) and low levels of free triiodothyronine (FT3) at < 0.39 pg/mL (normal range: 2.3–4.0.3.0) and free thyroxine (FT4) at < 0.04 pg/mL (normal range: 0.9–1.7), ACTH < 1.5 pg/mL and cortisol 2.04 µg/dL. Thyroid hormone replacement therapy was initiated, and steroid replacement therapy was continued, based on a diagnosis of hypothyroidism due to irAE-induced hypopituitarism. After the patient’s symptoms improved, nivolumab was administered as an adjuvant therapy for one year. The patient continues to be treated with steroids and thyroid hormones and has been alive for two years without recurrence.

### Case 2

**Fig. 2:**
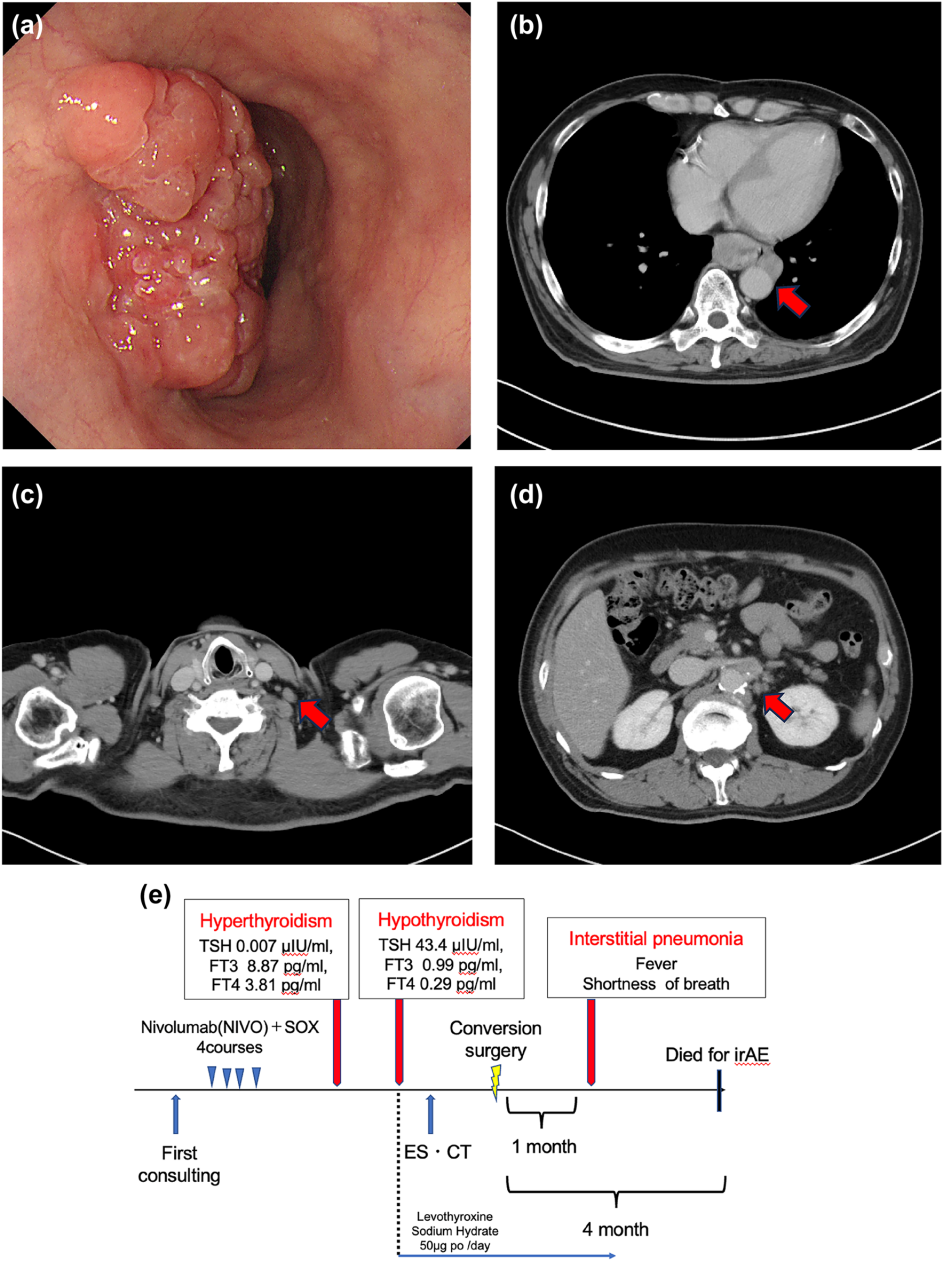

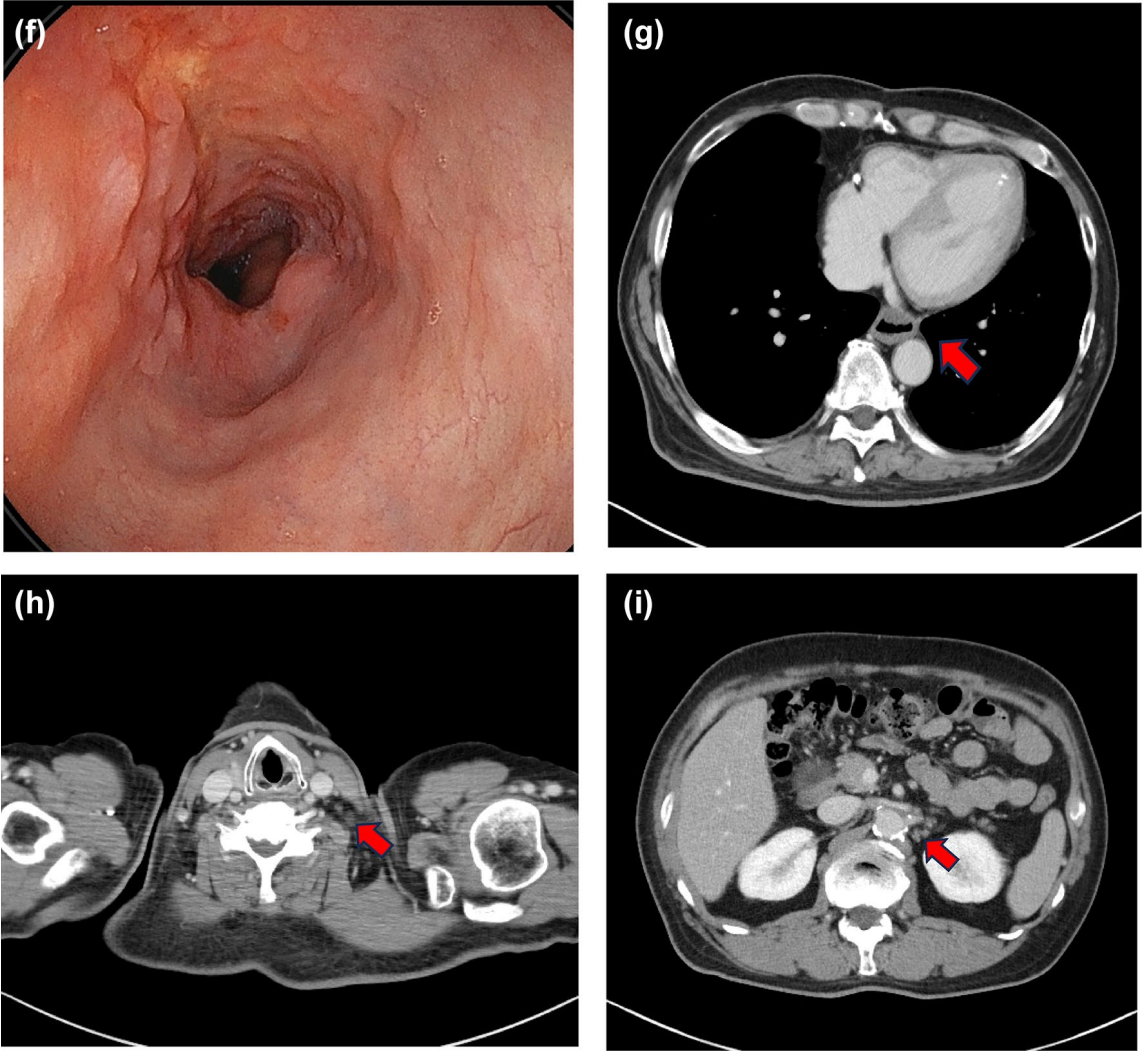
Imaging for Case 2: Initial EGD showing a Type 2 lesion around the EGJ with 8 cm esophageal invasion (**a**). CT showing enlarged #112-aoA LNs invading the thoracic aorta (**b**), #104L (**c**), and #16 LNs (**d**). Treatment course (**e**). EGD showing marked shrinkage of the primary tumor (**f**) and CT showing release of the aortic invasion due to #112aoA LN shrinkage (**i**) and #104L LN (**g**) and #16 LN (**h**) shrinkage after the induction of ICI-combined chemotherapy. EGD, esophagogastroduodenoscopy; CT, computed tomography; LN, lymph node; ICI, immune checkpoint inhibitor

A 65-year-old male presented with staggering anemia. The patient had a history of post-coronary artery bypass grafting (CABG) for angina pectoris. EGD showed a Siewert type I tumor with 8 cm esophageal invasion (Fig. 2a). Histopathological examination of a biopsy specimen resulted in a diagnosis of adenocarcinoma, and HER2-negative due to HER2 IHC 2+/FISH -. CT revealed wall thickening from the lower thoracic esophagus to the EGJ. In addition, CT showed enlarged mediastinal LNs (LN station #112-aoA) invading the thoracic aorta, left supraclavicular LNs (LN station #104L), and para-abdominal aortic LNs (LN station #16), which were considered unresectable (Fig. 2b–d). Multiple mediastinal and paragastric LNs were also observed on CT. The clinical diagnoses were cT4b (#112aoA-Ao), N3 (#106rR, #112aoA, #1, #3, and #7), M1 (#104L and #16), and Stage IVB.

**Fig. 3:**
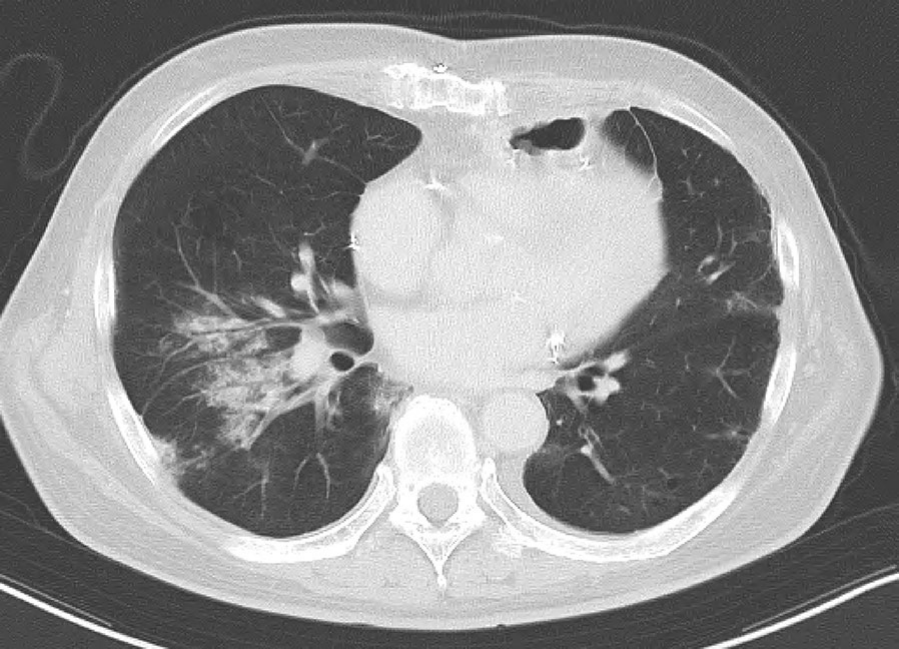
CT showing localized ground-glass opacities in both lungs at admission in Case 2. CT, computed tomography

**Fig. 4:**
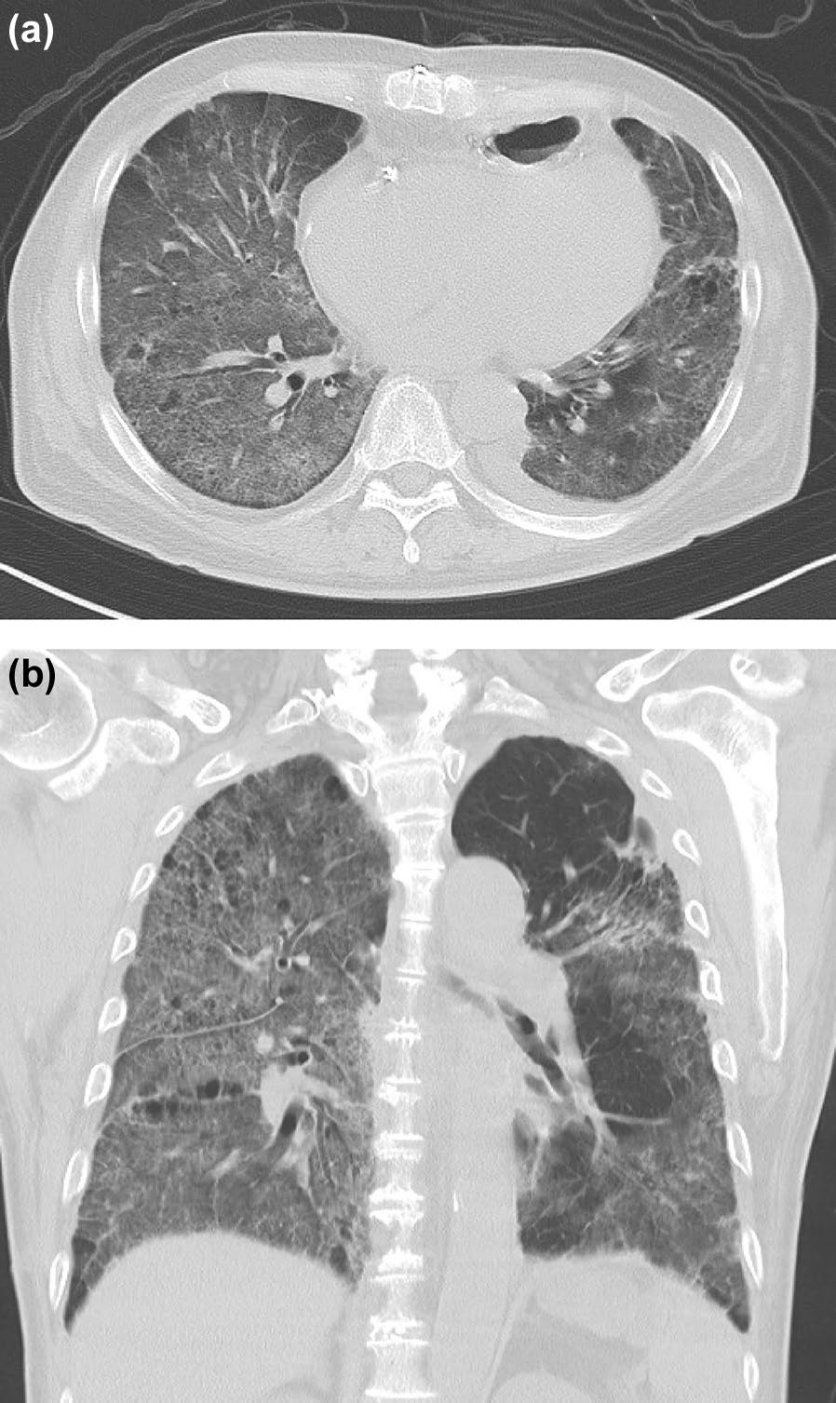
CT showing extensive interstitial shadows in both lungs following worsening respiratory condition after hospitalization in Case 2. **a**: coronal. **b**: axial. CT, computed tomography

After four courses of Nivo + SOX, the patient’s blood test findings included low level of TSH at 0.007 µIU/mL and high levels of FT3 at 8.87 pg/mL and FT4 at 3.81 pg/mL, indicating hyperthyroidism (Fig. 2e). Shortly thereafter, a follow-up blood test showed high level TSH at 6.71 µIU/mL and low levels FT3 at 0.99 pg/mL and FT4 at 0.29 pg/mL, a shift to hypothyroidism likely caused by thyroid gland destruction due to irAEs. Thyroid hormone replacement therapy was initiated. Imaging studies after Nivo + SOX therapy showed marked shrinkage of the primary tumor and LNs, including LN stations #104L and #16 (Fig. 2f, h, i). The #112aoA LN showed major shrinkage, resolution of the aortic invasion, and tumor downstaging (Fig. 2g). Conversion surgery was planned and thoracoscopic esophagectomy with three field lymph node dissection was safely performed. The operation time was 541 min, with an intraoperative blood loss of 128 mL. The pathological diagnosis was ypT0 (small number of residual carcinoma cells) N0M0, Stage 0 (UICC 8th ed.), with a histological therapeutic effect of grade 2b. The patient had an uneventful postoperative course and was discharged on postoperative day 22. However, four days after discharge, the patient visited the emergency room due to fever and respiratory distress. CT revealed localized ground-glass opacities in both lungs (Fig. 3). The percutaneous arterial oxygen saturation (SpO2) was 98% on room air, and C-reactive protein (CRP) was 6.76 mg/dL indicating mildly elevated inflammatory markers. The patient was admitted to the hospital urgently for suspected aspiration pneumonia. Antibiotic therapy was started, but on day 5 of treatment, SpO2 dropped to 94% despite 4 L oxygen administration, indicating a sudden increase in oxygen demand. The inflammatory values worsened as CRP 19.88 mg/dL, and a follow-up CT showed extensive interstitial shadows in both lungs (Fig. 4a, b). The laboratory date for interstitial pneumonia revealed was high as SP-D 245 ng/mL (normal range: <110) and ferritin 454 ng/mL (normal range: 13–277). Based on clinical suspicion of irAEs interstitial pneumonitis, steroid pulse therapy was initiated immediately, but due to worsening respiratory status, cyclophosphamide therapy was also started. Despite escalation to mechanical ventilation and extracorporeal membrane oxygenation (ECMO), the patient’s respiratory condition continued to worsen. Consequently, he died four months after conversion surgery.

## Discussion and conclusions

In recent years, the number of cases of EGJ adenocarcinoma in Asia has been increasing, possibly due to the westernization of diet, reflux esophagitis, and obesity [5]. EGJ cancer is a borderline lesion between the esophagus and stomach. In recent years, the standard surgical procedure involving regional LN dissection has been decided according to the length of esophageal invasion [6, 7]. In drug therapy, personalized treatment using biomarkers is advancing. Beyond the established use of trastuzumab in HER2-positive cases, new molecularly targeted drugs are being applied based on claudin 18 expression, while CPS and MSI measurements serve as indicators for selecting ICI therapy [8]. Clinical trials have demonstrated the efficacy of ICI-combined chemotherapy as a first-line treatment for unresectable EGJ cancer [9, 10]. In addition, the oncological benefits of conversion surgery after induction chemotherapy for originally unresectable EGJ cancers have been reported [11]. Furthermore, the use of neoajuvant chemoimmunotherapy, including ICI, may become treatment option in the future [12]. Consequently, the frequency of cases undergoing ICI treatment with surgery as a prerequisite, not just conversion surgery, may increase. However, perioperative safety in patients treated with ICI-combined chemotherapy followed by conversion surgery remains unknown [13].

An irAE is a series of adverse events resembling an autoimmune reaction that occurs in patients receiving ICIs [14]. The reported incidence of all grades of irAEs is 72% for programmed cell death protein 1 (PD-1) antibodies [15].

IrAEs occur not only during but also after treatment. Delayed-onset irAEs, occurring three months or more after the completion of ICI treatment, have recently received much attention [16]. Observational studies using the World Health Organization (WHO) global database of Individual Case Safety Reports (ICSRs) [17] tended to report mainly severe irAEs, although delayed-onset irAEs were reported in 5.7% of all cases. The most frequently reported delayed-onset irAEs are thyroiditis, pneumonitis, interstitial lung disease, hepatitis, and vitiligo. The median time to onset from the completion of ICI treatment is 167 days (approximately six months).

Additionally, early-onset irAEs might be a risk factor for the development of delayed-onset irAEs, with 11.1% of delayed-onset irAEs occurring as early events. Comparison of early- and delayed-onset irAEs showed involvement of different organs in 60.5% of patients, highlighting the need to consider delayed irAEs in previously unaffected sites.

The times from ICI therapy completion to the onset of delayed irAEs in the first and second cases in the present report were six and three months, respectively. In both cases, early onset irAEs, which might be a risk factor for delayed-onset irAEs, occurred preoperatively, and delayed-onset irAEs occurred postoperatively.

Whether surgical invasiveness is associated with the occurrence of delayed irAEs remains unclear; however, relatively invasive surgical procedures can affect patient immune status and could potentially be directly influenced by specific surgical manipulation. In case 1, hypothyroidism occurred as delayed irAE. It cannot be ruled out that the surgical procedure itself may have affected the immune system. In case 2, interstitial pneumonia may be influenced by the surgical procedure itself, but we also consider it highly likely that thoracic manipulation was involved. These cases taught us the necessity of always considering both the indirect effects of surgical invasiveness and the direct effects of surgical manipulation. As the number of conversion surgeries for EGJ cancer is expected to increase owing to the effectiveness of ICIs, the risk of postoperative delayed irAEs requires increased attention.

The organs most affected by ICI were the respiratory and Gastrointestinal, endocrine systems and did not differ between early- and delayed-onset irAEs [17]. In both cases, the organs in which early and delayed irAEs occurred differed. Thus, irAEs that may have been latent preoperatively may have developed after the surgical intervention.

The risk factors for irAEs were categorized into three groups: patient-related medical history, primary tumor histology, and ICI agents. Among patient factors, Eastern Cooperative Oncology Group performance status (PS) ≥ 2, body mass index (BMI) ≥ 25 kg/m^2^, sarcopenia, and chronic smoking increase the risk of interstitial lung disease [18]. Pneumonia and myocarditis are often reported as grade IV–V severe irAEs and occur early during ICI treatment. The predictive factors for very severe pneumonia and myocarditis include elevated neutrophil-to-lymphocyte ratio, PS ≥ 2, and albumin < 3.5 mg/dL [19].

Anti-PD-1 antibodies are associated with high incidences of pneumonitis, hypothyroidism, arthralgia, and vitiligo [20]. Although much of the existing literature has focused on irAEs during mono or dual ICI therapy, combination regimens with chemotherapy—now standard in many cancers—appear to further increase the incidence of pneumonitis [21]. Both patients in the present report had a history of chronic smoking and received ICI-based combination chemotherapy, placing them at increased risk. One patient ultimately developed fatal interstitial pneumonitis.

Although the precise mechanisms of irAEs have not been fully investigated, cytotoxic T cell activation by ICI may cause significant inflammation in organs other than tumor cells [22]. ICIs also remain in the patient for a long time; therefore, even after discontinuing ICI administration, T cell activity may increase and cause delayed-onset irAEs [23].

These two cases demonstrate that irAEs can occur even after conversion surgery and can be fatal. If we had been aware of the severe delayed-onset irAE, Case 2 could have been saved. Case 2, who developed pneumonitis, initially showed few typical interstitial shadows and was treated for aspiration pneumonia, which is likely to occur after surgery for EGJ cancer. In addition to the surgical invasiveness, aspiration pneumonia may have triggered the irAE-related interstitial pneumonia. Since Case 2 was a high-risk patient for delayed-onset irAE, interstitial pneumonia related tests should have been performed and steroid therapy initiation considered at the time treatment began for aspiration pneumonia. Patients at high risk of delayed-onset irAEs should be fully informed of this risk following conversion surgery. Moreover, irAEs should be suspected in these patients and treatment should be initiated as soon as symptoms appear.

In conclusion, intensive care for delayed-onset irAEs is needed in patients with initially unresectable EGJ cancer undergoing conversion surgery following ICI combined with chemotherapy treatment.

## Data Availability

No datasets were generated or analysed during the current study.

## Abbreviations

irAE: Immune-related adverse event
ICI: Immune checkpoint inhibitor; EGJ, esophagogastric junction
ECMO: Extracorporeal membrane oxygenation
CT: Computed tomography; EGD; esophagogastroduodenoscopy
PS: Eastern Cooperative Oncology Group performance status
BMI: Body mass index

## References

[1] Huang S, Wang S, Gao Z, Li Z, Wu H, Xu W, et al. Induction immunochemotherapy yields a higher conversion rate and better overall survival than chemotherapy in initially unresectable esophageal squamous cell carcinoma. Ann Surg Oncol. 2024;31:6635–44. 10.1245/s10434-024-15458-8. 38796589 10.1245/s10434-024-15458-8

[2] Huang S, Wu H, Cheng C, Zhou M, Xu E, Lin W, et al. Conversion surgery following immunochemotherapy in initially unresectable locally advancer esophageal squamous cell carcinoma – a real–world multicenter study (RICE retro). Front Immunol. 2022;13:935374. 10.3389/fimmu.2022.935374. 35911702 10.3389/fimmu.2022.935374 PMC9326168

[3] Mine S, Tanaka K, Kawachi H, Shirakawa Y, Kitagawa Y, Toh Y, et al. <article-title update="added">Japanese Classification of Esophageal Cancer, 12th Edition: Part I. Esophagus. 2024;21(3):179–215. 38568243 10.1007/s10388-024-01054-y PMC11199297

[4] Brieleyt JD, Gospodarowicz MK, Wittekind C. TNM classification of Malignant Tumors International Union Against Cancer. 8th ed. Oxford, England: John Willey & Sons; 2017. p. 57–62.

[5] Matsuno K, Ishihara R, Ohmori M, Iwagami H, Shichijyo S, Maekawa A, et al. Time trends in the incidence of esophageal adenocarcinoma, gastric adenocarcionoma, and superficial esophagogastric junction adenocarcinoma. J Gastroenterol. 2019;54:784–91. 10.1007/s00535-019-01577-7. 30927083 10.1007/s00535-019-01577-7

[6] Kitagawa Y, Ishihara R, Ishikawa H, Ito Y, Oyama T, Oyama T, et al. Esophageal cancer practice guidelines 2022 edited by the Japan esophageal society: part 2. Esophagus. 2023;20:373–89. 10.1007/s10388-023-00994-1. 36995449 10.1007/s10388-023-00994-1 PMC10235142

[7] Koyanagi K, Kato F, Kanamori J, Daiko H, Ozawa S, Tachimori Y. Clinical significance of esophageal invasion length for the prediction of mediastinal lymph node metastasis in Siewert type II adenocarcionoma retrospective single-institution study. Ann Gastroenterol Surg. 2018;2:187–96. 10.1002/ags3.12069. 29863189 10.1002/ags3.12069 PMC5980392

[8] Bang YJ, Van Cutsem E, Feyereislova A, Chung HC, Shen L, Sawaki A, et al. Trastuzumab in combination with chemotherapy versus chemotherapy alone for treatment of HER2-positive advanced gastric or gastro-oesophageal junction cancer (ToGA): a phase 3, open-label, randomised controlled trial. Lancet. 2010;376(9742):687–97. 20728210 10.1016/S0140-6736(10)61121-X

[9] Kang YK, Chen LT, Ryu MH, Oh DY, Oh SC, Chung HC, et al. Nivolumab plus chemotherapy versus placebo plus chemotherapy in patients with HER2-negative, untreated, unresectable advanced or recurrent gastric or gastro-oesophageal junction cancer (ATTRACTION-4): a randomised, multicentre, double-blind, placebo-controlled, phase 3 trial. Lancet Oncol. 2022;23:234–47. 10.1016/S1470-2045(21)00692-6. 35030335 10.1016/S1470-2045(21)00692-6

[10] Janjigian YY, Shitara K, Moehler M, Garrido M, Salman P, Shen L, et al. First-line nivolumab plus chemotherapy versus chemotherapy alone for advanced gastric, gastro-oesophageal junction, and oesophageal adenocarcinoma (CheckMate 649): a randomised, open-label, phase 3 trial. Lancet. 2021;398:27–40. 10.1016/S0140-6736(21)00797-2. 34102137 10.1016/S0140-6736(21)00797-2 PMC8436782

[11] Yoshida K, Yasuhuku I, Terashima M. International retrospective cohort study of conversion therapy for stage IV. Gastric cancer 1 (CONVO-GC-1). Ann Gastroenterol Surg. 2021;6:227–40. 35261948 10.1002/ags3.12515 PMC8889854

[12] Liu X, Ma B, Zhao L. Neoadjuvant chemoimmunotherapy in locally advanced gastric or gastroesophageal junction adenocarcinoma. Front Oncol. 2024;14:1342162. 38686192 10.3389/fonc.2024.1342162 PMC11056579

[13] Shoji Y, Kanamori K, Koyanagi K, Otsuka T, Nakashima R, Tajima K, et al. Conversion surgery for esophageal and esophagogastric junction cancer. Int J Clin Oncol. 2024;29:1777–84. 10.1007/s10147-024-02639-4. 39436571 10.1007/s10147-024-02639-4 PMC11588808

[14] Brahmer JR, Lacchetti C, Schneider BJ, Atkins MB, Brassil KJ, Caterino JM, et al. Management of immune-related adverse events in patients treated with immune checkpoint inhibitor therapy: American Society of Clinical Oncology clinical practice guideline. J Clin Oncol. 2018;36:1714–68. 10.1200/JCO.2017.77.6385. 29442540 10.1200/JCO.2017.77.6385 PMC6481621

[15] Song P, Zhang D, Cui X, Zhang L. Meta-analysis of immune-related adverse events of immune checkpoint inhibitor therapy in cancer patients. Thorac Cancer. 2020;11:2406–30. 10.1111/1759-7714.13541. 32643323 10.1111/1759-7714.13541 PMC7471041

[16] Naidoo J, Murphy C, Atkins MB. Society for cancer (SITC) consensus definitions for immune checkpoint inhibitor-associated immune-related adverse events (irAEs) terminology. J Immunother Cancer. 2023;11:e006398. 37001909 10.1136/jitc-2022-006398 PMC10069596

[17] Noseda R, Bedussi F, Giunchi V, Fusaroli M, Raschi E, Ceschi A. Reporting of late-onset immune-related adverse events with immune checkpoint inhibitors in vigibase. J Immunother Cancer. 2024;12:e009902. 10.1136/jitc-2024-009902. 39489542 10.1136/jitc-2024-009902 PMC11535709

[18] Chennamadhavuni A, Abushahin L, Jin N, Presley CJ, Manne A. Risk factors and biomarkers for immune-related adverse events: a practical guide to identifying high-risk patients and rechallenging immune checkpoint inhibitors. Front Immunol. 2022;13:779691. 10.3389/fimmu.2022.779691. 35558065 10.3389/fimmu.2022.779691 PMC9086893

[19] Ruste V, Goldschmidt V, Laparra A, Messayke S, Danlos FX, Romano-Martin P, et al. The determinants of very severe immune- related adverse events associated with immune checkpoint inhibitors: a prospective study of the French REISAMIC registry. Eur J Cancer. 2021;158:217–24. 10.1016/j.ejca.2021.08.048. 34627664 10.1016/j.ejca.2021.08.048

[20] Khoja L, Day D, Wei-Wu Chen T, Siu LL, Hansen AR. Tumour- and class-specific patterns of immune-related adverse events of immune checkpoint inhibitors: a systematic review. Ann Oncol. 2017;28:2377–85. 10.1093/annonc/mdx286. 28945858 10.1093/annonc/mdx286

[21] Fujiwara Y, Horita N, Namkoong H, Galsky MD. The effect of adding immune checkpoint inhibitors on the risk of pneumonitis for solid tumours: a meta-analysis of phase III randomised controlled trials. Eur J Cancer. 2021;150:168–78. 10.1016/j.ejca.2021.03.012. 33906099 10.1016/j.ejca.2021.03.012

[22] Passat T, Touchefeu Y, Gervois N, Jarry A, Bossard C, Bennouna J. Physiopathological mechanisms of immune-related adverse events induced by anti-CTLA-4, anti-PD-1 and anti-PD-L1 antibodies in cancer treatment. Bull Cancer. 2018;105:1033–41. 10.1016/j.bulcan.2018.07.005. 30244981 10.1016/j.bulcan.2018.07.005

[23] Fukudo M, Sasaki T, Ohsaki Y. PD-1 blockers: staying long in the body and delayed toxicity risks. J Thorac Oncol. 2020;15:e42–4. 10.1016/j.jtho.2019.11.015. 32093860 10.1016/j.jtho.2019.11.015

